# Solitary epidural lipoma

**DOI:** 10.11604/pamj.2018.30.52.15154

**Published:** 2018-05-21

**Authors:** Omar Boulahroud, Tarik Ziadi

**Affiliations:** 1 Department of Neurosurgery, Military Hospital My Ismail, Meknes, Morocco; 2 Department of Radiology, Military Hospital My Ismail, Meknes, Morocco

**Keywords:** Epidural lipoma, spine, obesity

## Image in medicine

Spinal epidural lipomatosis (SEL) is defined as normal adipose tissue that is pathologically overgrown in the epidural space of the spinal canal. It usually occurs in obese people, patients with history of corticosteroid use or endocrinopathy. They usually present with gradually progressing symptoms and are rarely associated with rapidly aggravated neurologic deficits. Radiologic assessments are mostly conducted by using MRI. A 65-year-old woman presented with isolated low back pain. She had no history of corticosteroid use, endocrinopathy or radicular pain. On examination, she was obese (height 155cm, weight 87kg) with a body mass index (BMI) of 56.1 kg/m^2^. Neurological exam was normal with no motor weakness and sensation was intact. Plain radiographs showed no evidence of abnormality, including instability and other pathologic findings. Magnetic resonance imaging (MRI) revealed an epidural mass posterior to the L5 vertebral body that was isosignal to subcutaneous fat and it compressed asymmetrically on the right side of the cauda equina and the exiting right L5 nerve root on sagittal (A) and axial (B) T2 weighted images. There was no evidence of spinal stenosis. The result of electromyography was normal. We recommended surgical intervention to relieve pain but the patient declined. Opioid analgesia has been prescribed and her symptoms became much improved. Weight reduction by physiotherapy and exercise resulted in complete loss of lumbar pain (the patient lost 30kg in 6 months) and MRI control showed a complete disappearance of the mass.

**Figure 1:**
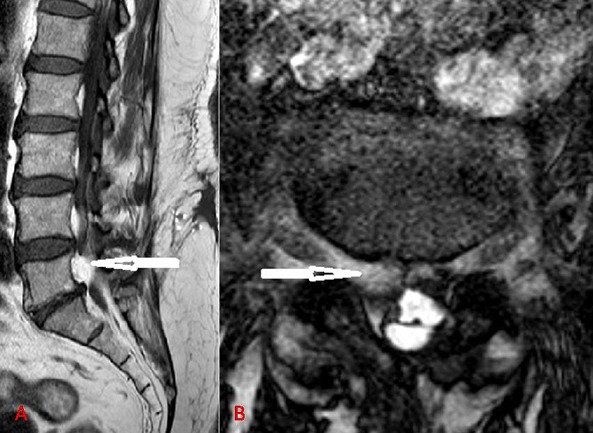
A) T2-weighted magnetic resonance (MR) sagittal images of the right paracentral area showing a mass (arrow) in the spinal canal at L5; B) T2-weighted axial magnetic resonance images at the L5-S1 neural foramen level

